# Assessing the short term impact of air pollution on mortality: a matching approach

**DOI:** 10.1186/s12940-017-0215-7

**Published:** 2017-02-10

**Authors:** Michela Baccini, Alessandra Mattei, Fabrizia Mealli, Pier Alberto Bertazzi, Michele Carugno

**Affiliations:** 1 Department of Statistics, Informatics, Applications “G. Parenti”, Università di Firenze, Viale Morgagni 59, 50134 Florence, Italy; 2 Department of Clinical Sciences and Community Health, Università degli Studi di Milano, Milan, Italy; 3 Epidemiology Unit, Department of Preventive Medicine, Fondazione IRCCS Ca’ Granda - Ospedale Maggiore Policlinico, Milan, Italy

**Keywords:** Air pollution, Attributable deaths, Causal inference, Health impact assessment, Mortality, Potential outcomes, Propensity score, Matching, Short term effect, Unconfoundedness

## Abstract

**Background:**

The opportunity to assess short term impact of air pollution relies on the causal interpretation of the exposure-response association. However, up to now few studies explicitly faced this issue within a causal inference framework. In this paper, we reformulated the problem of assessing the short term impact of air pollution on health using the potential outcome approach to causal inference. We considered the impact of high daily levels of particulate matter ≤10 μm in diameter (PM_10_) on mortality within two days from the exposure in the metropolitan area of Milan (Italy), during the period 2003–2006. Our research focus was the causal impact of a hypothetical intervention setting daily air pollution levels under a pre-fixed threshold.

**Methods:**

We applied a matching procedure based on propensity score to estimate the total number of attributable deaths (AD) during the study period. After defining the number of attributable deaths in terms of difference between potential outcomes, we used the estimated propensity score to match each high exposure day, namely each day with a level of exposure higher than 40 μg/m^3^, with a day with similar background characteristics but a level of exposure lower than 40 μg/m^3^. Then, we estimated the impact by comparing mortality between matched days.

**Results:**

During the study period daily exposures larger than 40 μg/m^3^ were responsible for 1079 deaths (90% CI: 116; 2042). The impact was more evident among the elderly than in the younger age classes. Exposures ≥ 40 μg/m^3^ were responsible, among the elderly, for 1102 deaths (90% CI: 388, 1816), of which 797 from cardiovascular causes and 243 from respiratory causes. Clear evidence of an impact on respiratory mortality was found also in the age class 65–74, with 87 AD (90% CI: 11, 163).

**Conclusions:**

The propensity score matching turned out to be an appealing method to assess historical impacts in this field, which guarantees that the estimated total number of AD can be derived directly as sum of either age-specific or cause-specific AD, unlike the standard model-based procedure. For this reason, it is a promising approach to perform surveillance focusing on very specific causes of death or diseases, or on susceptible subpopulations. Finally, the propensity score matching is free from issues concerning the exposure-confounders-mortality modeling and does not involve extrapolation. On the one hand this enhances the internal validity of our results; on the other, it makes the approach scarcely appropriate for estimating future impacts.

## Background

Since the year 2000, many epidemiological studies have quantified short term and long term impacts of air pollution on health in terms of the number of health events due to air pollutant exposures exceeding pre-fixed thresholds [1–5]. Short term impacts, i.e. the impacts observed within few days from the exposure, provide only a partial picture of the health damage attributable to air pollution, because they do not consider consequences of long term exposures, that are characterized by much stronger associations [6–8]. However, assessing short term impacts has the advantage of allowing an appraisal of the air pollution effect that is not affected by issues that are critical in long term evaluation, such as latency time definition and cumulative exposure assessment [9]. Also, short term impacts stress the beneficial effect of measures targeted to immediately improve air quality.

The standard approach to estimate the short term impact of air pollution relies on regression methods. Focusing on mortality, first, the curve describing the relationship between daily exposure and daily deaths is estimated through a Poisson regression model, adjusting for possible confounders; then, the estimated curve is combined with the observed mortality and air pollutant levels to calculate how many of the observed deaths are attributable to the exposures exceeding a fixed threshold.^1^ Varying the threshold, different hypothetical scenarios of air pollution reduction are defined and the impact due to exceeding national or international air quality standards, or limits recommended by agencies for public health protection is quantified [10]. The shape of the exposure-response function is usually assumed to be log-linear [11–14].

The opportunity to assess the short term impact of air pollution relies on the causal interpretation of the exposure-response association. Up to now, this causal interpretation has been mainly supported by the fact that studies carried out in different countries and contexts provided consistent findings. Moreover, especially for airborne particulate matter, the evidence on the biological mechanisms tying exposure and health damage is consolidated, substantiating the plausibility of the observed associations [15]. Bellini et al. [16] read short term effect estimates in light of the Bradford Hill causation criteria [17], showing that they were largely fulfilled. However, also Hill made it explicit that decisions about cause-effect relations cannot be based on a set of rules [18]. The principal limitation of this reasoning is the lack of a formal and rigorous definition of causal effect and of the explicit definition of the assumptions needed for a causal interpretation of the epidemiological evidence [19–21].

The potential outcome approach to causal inference, commonly referred to as the Rubin’s Causal Model (RCM) [22, 23], encourages thinking in terms of causes and action’s consequences, within a formal mathematical framework. Despite its increasing popularity in many fields, including epidemiology and medical sciences, to the best of our knowledge it is relatively new in studies aimed at assessing the effects and the impact of air pollution on health. Wang et al. [24] addressed confounding adjustment in model-based estimation of the exposure-response relationship, arguing that their approach is related to causal inference although they do not take a causal inference perspective. They applied their method to daily time series data in order to estimate the short-term effects of air pollution on emergency admissions for cardiovascular diseases in Nassau, NY. In a short commentary to their paper, Gutman and Rubin [25] suggested the use of RCM to estimate the causal effect of air pollution. However, they provided only a theoretical scheme for inference, without any example on real data. More recently, Zigler and Dominici [26] discussed the potential contribution of the potential outcome approach in the policy debate about air pollution regulatory interventions, and provided a classification to frame the studies in this field. In a second work, the same authors provided new analytic perspectives and statistical methods for drawing causal inferences on the long-term health effects of air quality regulations [27, 28]. They assessed causal effects of an actual intervention, which had the reduction of fine air born particles in the atmosphere and, thereby, improvement in health as outcomes; specifically, their aim was to disentangle causal effects of the intervention both through and not through the reduction of the air pollutant levels. An attempt to use the potential outcome approach to assess the short term effect of air pollution on mortality can be found also in Schwartz et al. [29]. They estimated the percent variation in mortality per increase in fine particles level, using two procedures which provided similar results. In the first procedure the estimate was obtained by stratifying days according to a score which summarized the distribution of the observed covariates (this score was different from the generalized propensity score used for continuous treatments [30]); the second procedure, which was applied in another recent paper as well [31], was based on an instrumental variable approach. Both these procedures are substantially different from the one we propose here.

In this paper, we reformulated the problem of assessing the short term impact of air pollution on health within the potential outcome approach to causal inference. We assessed the causal impact, expressed in terms of attributable deaths (AD), of high daily levels of particulate matter ≤ 10 μm in diameter (PM_10_) on mortality in the metropolitan area of Milan (Italy), during the period 2003–2006. Specifically, we compared the number of deaths we observed in days with exposure levels higher than 40 μg/m^3^ with the number of deaths that we would have observed if in all those days exposure levels had been lower than 40 μg/m^3^. Fixing the threshold to 40 μg/m^3^ assured that the resulting counterfactual time series largely respected the limit of 40 μg/m^3^ for PM_10_ annual average, which defines the legal obligation for European Union member states [32]. According to the classification proposed by Zigler and Dominici [26], the present work can be framed as a study aimed to evaluate the causal impact of a hypothetical intervention setting daily exposure levels under a pre-fixed threshold (assuming that the effect of this hypothetical intervention would occur only through the reduction of PM_10_ levels). This interpretation allows to relate causal effects to regulatory standards. It is worth to notice that assessing the impact of a hypothetical intervention setting daily exposure levels under 40 μg/m^3^ is different from assessing the impact (by using an exposure-response function) setting the counterfactual exposure to a specific level, e.g. 40 μg/m^3^, i.e. implicitly assuming an intervention, difficult to conceive, which is able to reduce daily exposure levels exactly to 40 μg/m^3^.

An impact evaluation on the same city and period has been previously conducted following a standard procedure by Baccini et al. [10].

## Methods

### Data

We considered data for the city of Milan for the years 2003–2006. Milan (1,299,633 inhabitants in 2007) is the capital city of the Lombardy region, in northwestern Italy. It is located in the basin of the Po River, an area characterized by unfavorable geographical and climate conditions which induce frequent phenomena of thermal inversion. As a consequence, air pollution, mainly deriving from road transport, is trapped close to the ground and reaches very high daily concentrations.

The air quality monitoring network of the Regional Environmental Protection Agency provided daily measurements of PM_10_, temperature, and relative humidity in the city. A unique daily time series of PM_10_ levels was obtained by averaging data over the available monitors [10]. According to large part of the literature, there exists an immediate effect of exposure on mortality which diminishes in few days [33, 34]. Therefore, in order to allow for comparison with previous results as well, we used the average of the current-day and previous-day PM_10_ concentrations (lag 0–1) as exposure variable.

Death certificates were obtained from the Regional Mortality Register. We focused on deaths of the resident population occurring inside the city area. We considered daily mortality from all (except for external) causes (International Classification of Diseases, Ninth Revision, codes below 800), and mortality by cardiovascular diseases (ICD-9: 390–459) and respiratory diseases (ICD-9: 460–519), separately. Daily mortality counts were classified by age groups: 15–64 years, 65–74 years, ≥75 years.

### Notation

Indicating with *X*_*i*_ the lag 0–1 exposure in day *i*, *i* = 1, …, *N*, we defined the treatment indicator *W*_*i*_, equal to 1 if *X*_*i*_ ≥ 40 μg/m^3^ (high exposure level) and zero otherwise (low exposure level). Then, according to the RCM, under the Stable Unit Treatment Value Assumption (SUTVA) [23], we associated to each day two potential outcomes: *Y*_*i*_(1), the number of deaths in *i* if exposure in *i* was ≥40 μg/m^3^, and *Y*_*i*_(0), the number of deaths in *i* if exposure in *i* was < 40 μg/m^3^. Obviously, we could only observe at most one of these potential outcomes for each day. Let *Y*_*i*_^*obs*^ denote the observed count of deaths in *i*: *Y*_*i*_^*obs*^ = *Y*_*i*_(0) if *W*_*i*_ = 0, and *Y*_*i*_^*obs*^ = *Y*_*i*_(1) if *W*_*i*_ = 1. We refer to days with *W*_*i*_ = 1 as “treated days” and to days with *W*_*i*_ = 0 as “control days”.

### Definition of attributable deaths

For each *i*, we defined the day-level AD as the difference between the two potential outcomes:

1 $$ A{D}_i={Y}_i(1)-{Y}_i(0). $$

Since we were interested in the total impact exposures ≥ 40 μg/m^3^ observed during the study period, we focused on treated days only and defined the total number of AD during the study period as the sum of the day-level impacts in equation 1 for *W*_*i*_ = 1:

2 $$ AD={\displaystyle \sum_i{W}_i\left({Y}_i(1)-{Y}_i(0)\right)}={\displaystyle \sum_i{W}_i} A{D}_i. $$

Being *Y*_*i*_(0) always missing in equation 2, in order to estimate *AD* we applied a matching procedure to impute these missing potential outcomes: for each treated day *i*, we found one control day with similar background characteristics (*matched control day*), and we used the mortality level observed in this day to impute *Y*_*i*_(0). We based our matching procedure on the propensity score, clearly specifying the underlying assumptions.

### Design phase: propensity score matching

Using a matching procedure requires the definition of a distance measure between units. Especially when the number of covariates is high, a convenient distance measure is based on the propensity score [35]. Let **Z**_*i*_ be a vector of background variables for day *i*. We defined the propensity score as the day-level probability of observing an exposure ≥ 40 μg/m^3^, conditional on **Z**_*i*_:

3 $$ {e}_i= e\left({\mathbf{Z}}_i\right)= P\left({W}_i=1\left|{\mathbf{Z}}_i\right.\right). $$

According to Rosenbaum and Rubin [35], if there are no unobserved confounders (unconfoundedness condition) and if there is sufficient overlap in the distribution of the covariates between treated and control days (so that for each treated day we can find a control day with similar background characteristics), adjusting for (e.g. by matching on) the propensity score is sufficient to remove confounding. The two conditions mentioned above define the strong ignorability assumption.^2^ A critical issue in the design phase of an observational study is us the choice of the background variables **Z**_*i*_ conditionally on which strong ignorability is reasonable. We based this selection on a priori substantive knowledge of the phenomenon derived from the literature on short term effects of air pollution, which suggests that the air pollution-mortality relationship can be confounded by meteorological conditions, short and long term seasonality and other factors that could produce unusual peaks of mortality.

#### Propensity score estimation

The propensity score for each unit was estimated from a logistic model for *W*_*i*_, including terms for all the relevant background variables **Z**_*i*_ :

4 $$ {W}_i\sim Bernoulli\left({e}_i\right)\ \mathrm{logit}\left({e}_i\right)= f\left({\mathbf{Z}}_i,\boldsymbol{\upbeta} \right), $$

where ***β*** was a vector of unknown coefficients and *f* a general function of covariates and coefficients. Then, indicating with $\widehat{\boldsymbol{\upbeta}}$ the vector of the estimated coefficients, the estimated propensity score was obtained as:

5 $$ {\widehat{e}}_i=\frac{ \exp \left( f\left({\mathbf{Z}}_i,\widehat{\boldsymbol{\upbeta}}\right)\right)}{1+ \exp \left( f\left({\mathbf{Z}}_i,\widehat{\boldsymbol{\upbeta}}\right)\right)}. $$

Different specifications were possible for the model in equation 4 and some effort was needed to find an appropriate *f*. Being the propensity score a balancing score [35], the key criterion driving the specification of *f* consists in obtaining predicted values ${\widehat{e}}_i$ conditionally on which the covariates distribution is the same in the treated and matched control groups.

We assessed the balancing property for each covariate, under different choices of *f*, by using suitable measures of differences of the covariates between treated and control days [36]: visual inspection of the distributions before and after matching, and comparison of pre- and post-matching standardized mean differences^3^, when applicable. Regarding seasonality, we assessed the balance by comparing the distribution of the variable “day of year” between groups, accounting for its circular nature [37]. For this purpose, after converting days of year to angular measurements, we calculated, before and after matching, the nonparametric statistic proposed by Wheeler and Watson, which provides a measure of difference between two distributions in case of circular variable [38]. Taking into account that larger values of the statistic are indicative of larger discrepancies between the two distributions, we used them as descriptive measures of balance in “day of year” between treated and control/matched days. We did not check for the balance of the calendar year because the study period was limited to 4 years only. However, checking that earlier years are not matched with later ones and vice-versa could be important in longer time series.

The propensity score model specification that led to the best balance in covariates distributions included season-specific indicators of day of the week and holiday, an indicator of days with influenza epidemics, a cubic regression spline with 5 of freedom per year on the calendar day to account for medium and long term seasonality, and a bivariate smooth term for temperature at lag 0–3 and humidity, defined by the tensor product of two marginal thin plate regression splines with basis dimensions 5 and 3, respectively [39]. We also included in the model an indicator for days with temperature exceeding 28 °C to capture the possible effect of extreme heat episodes, and an indicator for the July-August period to account for the reduction in the number of city residents during summer holidays.

#### Nearest neighbor matching

For each treated day *i*, we selected as match the control day with estimated propensity score closest to *i* (*nearest neighbor matching*) [40]. We used matching with replacement, allowing for each control day to be used as a match more than once. Matching with replacement produces matches of higher quality than matching without replacement, reducing bias even at the cost of losing some precision [41].

### Analysis phase: AD estimation

For each treated day *i*, we first imputed the missing potential outcome *Y*_*i*_(0) using the count of deaths observed in its matched-control day *Y*_*i*_^*C*^. Then, we estimated the day-level impact for each treated day as the difference:

6 $$ {\widehat{AD}}_i={Y}_i^{obs}-{Y}_i^C. $$

Finally, we estimated *AD*:

7 $$ \widehat{AD}={\displaystyle {\sum}_i{W}_i{\widehat{AD}}_i}. $$

The estimate of the variance of $\widehat{AD}$ was derived from the sample variance reported in Abadie and Imbens [40] for the Sample Average effect of Treatment on the Treated estimator^4^.

All analyses were performed in R software (R Core Team; https://cran.r-project.org/).

## Results

In Milan between 2003 and 2006 there were, on average, 31.2 natural deaths per day, 10.3 from cardiovascular causes and 2.5 from respiratory causes. The annual average level of exposure (PM_10_ at lag 0–1) was 52.5 μg/m^3^; 812 days during the study period (55.7%) exceeded 40 μg/m^3^ and 593 days (40.7%) exceeded the daily limit of 50 μg/m^3^ which should not be exceeded more than 35 days per year according to the EU legislation [32]. The exposure levels were sometimes very high, with about 9% of days exceeding 100 μg/m^3^.

**Fig. 1:**
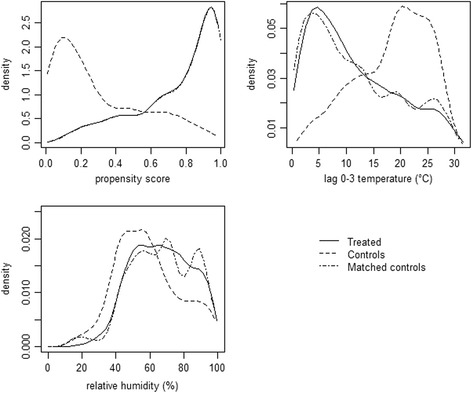
Density functions of estimated propensity score, average temperature in the current and in the previous three days (lag 0–3) and relative humidity for treated days, control days and matched control days, Milan, Italy, 2003–2006. Note that for propensity score the treated and matched curves are completely overlapping

Table 1 and Fig. 1 report the results of the balancing property check for the selected propensity score model. While the propensity score distributions in treated and control days were very different, the distribution in matched control days completely overlapped the distribution in treated days. Treated days were characterized by very different temperature and relative humidity with respect to control days. However, the distributions of the meteorological variables in the matched samples were similar, as confirmed by the standardized differences which were very small after matching. The percentage of heat episodes was similar among treated and control days both before and after matching. Days characterized by peaks of influenza were 12.8% in the treated group and only 0.9% in the control group. After matching, the standardized difference decreased by 34.7% but balancing remained unsatisfactory. This result deserves some consideration. In principle, influenza epidemics could be treated as an additional treatment rather than a covariate. In such a case, an assignment mechanism for a multivariate treatment should be specified, and a matching procedure based on a generalized propensity score approach for this multivariate treatment should be considered [30]. Being this extension beyond the scope of the paper, in order to investigate the role of influenza epidemics and check for the robustness of our results, we simply performed a sensitivity analysis by estimating the impact on the subset of days without influenza epidemics (see below).

**Table 1:** Covariates balance before and after matching, Milan, Italy, 2003–2006

	Mean/Proportion			Standardized difference^d^		
Background characteristic	Treated (*n* = 812)	Controls (*n* = 649)	Matched Controls (*n* = 649)	Pre-matching	Post-matching	% Bias^e^
Estimated propensity score	0.756	0.306	0.756	1.810	0	100.0
Temperature (°C)^a^	11.4	18.3	11.3	0.914	0.013	98.5
Relative humidity (%)	66.8	58.6	67.1	0.456	0.014	97.0
Saturdays and Sunday	0.243	0.341	0.195	0.217	0.106	51.0
Day of year	-	-	-	405.5^c^	15.9^c^	96.1^f^
Influenza epidemics	0.128	0.009	0.054	0.483	0.315	34.7
Heat episodes^b^	0.032	0.028	0.025	0.001	0.002	−77.8
Summer days	0.225	0.664	0.252	0.037	0.002	93.8

^a^Temperature: average temperature in the current and in the previous 3 days

^b^Heat episodes: days with temperatures exceeding 28 °C. Summer days: from May 1st to September 30th

^c^Wheeler and Watson’s statistics (W) for the comparison of the distribution of the circular variable “day of the year” between treated and control units (pre-matching W) or between treated and matched controls (post-matching W)

^d^Standardized difference: pre-matching (${\delta}^{pre}$) and post-matching (${\delta}^{post}$)

^e^% bias: $$ 100\times \left({\delta}^{pre}-{\delta}^{post}\right)/{\delta}^{pre} $$

^f^Percent reduction in the Wheeler and Watson’s statistics after matching

**Fig. 2:**
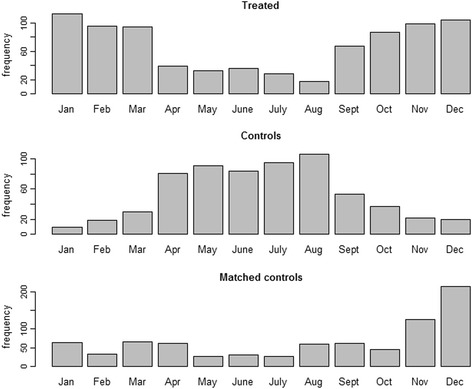
Distributions of treated days, control days and matched control days by calendar month, Milan, Italy, 2003–2006

In order to check balance for day of the week, we focused on the percentage of Saturdays and Sundays, which resulted very similar in treated and matched control days. In order to check seasonality balance, we focused on a warm season indicator, which was equal to 1 from May 1st to September 30th and 0 elsewhere, and on the variable day of year. Matching clearly reduced the percentage of summer days in favor of winter days (Fig. 2). Substantial balance after matching was found for the percentage of warm season days, with % bias reduction after matching equal to 93.8%. The Wheeler and Watson’s statistic, which was equal to 405.5 before matching, reduced by 96.1% after matching, indicating a substantial improvement of the balance in day of year.

**Fig. 3:**
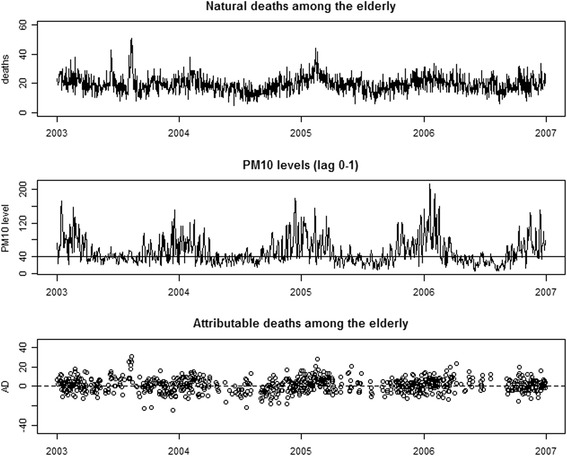
Daily counts of deaths among people aged 75 and over (upper panel), average PM_10_ level in the current and in the previous day (lag 0–1) (middle panel) and estimated daily attributable deaths (lower panel), Milan, Italy, 2003–2006

Figure 3 shows the daily number of natural deaths during the study period, together with daily exposures and day-level impacts by calendar day, among the elderly. Day-level impacts appeared to be rather heterogeneous. Exposures ≥ 40 μg/m^3^ were observed mainly during winter, but we estimated relevant positive day-level impacts also during summer, which could be indicative of a possible interaction between temperature and exposure. We could be surprised by the presence of negative estimates of the day-level impacts (Fig. 3). This might lead to misleading conclusions if we do not consider that each ${\widehat{AD}}_i$ depends on the imputed value of the missing potential outcome *Y*_*i*_(0). This imputation is naturally subject to variability, which may lead to negative estimated impacts, even for days where air pollution had indeed small or no impact. In this sense, negative ${\widehat{AD}}_i$ are fully consistent with the existence of a harmful effect of the exposure.

Table 2 shows the estimated AD for each cause of death and age class, along with their 90% confidence intervals. These results should be interpreted as the number of deaths that would have been avoided on average, if had the daily level of exposure never exceeded 40 μg/m^3^ during the study period. The impact was concentrated among individuals over 75. Exposures ≥ 40 μg/m^3^ were responsible, among the elderly, for 1102 deaths (90% CI: 388, 1816), of which 797 from cardiovascular causes (90% CI: 305, 1288) and 243 from respiratory causes (90% CI: −22, 508).

**Table 2:** Estimated number of attributable deaths by cause and age class, Milan, Italy, 2003–2006

	Age 15–64		Age 65–74		Age 75+		All ages (15+)	
	AD	90% CI	AD	90% CI	AD	90% CI	AD	90% CI
Cardiovascular causes	−172	−368, 24	91	−244, 426	797	305, 1288	716	117, 1315
Respiratory causes	−25	−133, 83	87	11, 163	243	−22, 508	305	17, 593
Other natural causes	153	−246, 552	−157	−401, 87	62	−414, 538	58	−496, 612
All natural causes	−44	−609, 521	21	−425, 467	1102	388, 1816	1079	116, 2042

*AD* attributable deaths, *90% CI* 90% confidence interval

Clear evidence of an impact of air pollution on respiratory mortality was found also in the age class 65–74, with 87 AD (90% CI: 11, 163). For the first age class (15–64) the confidence intervals were extremely wide, so that clear conclusions could not be drawn. It is worth noticing that the estimate of the total number of AD (1079) corresponded to the sum of the age- and cause-specific impacts; the confidence interval around this value was large, yet clearly far from zero (90% CI: 116, 2042).

The estimated AD from other causes were lower than the AD from cardiovascular and respiratory diseases (with the exception of the first age class), and the associated confidence intervals always included zero (the null hypothesis of no impact). This analysis on other causes of death, which can be interpreted as a proxy of a negative control analysis [42], showed that the estimated impacts would be negligible if deaths from cardiovascular and respiratory diseases were excluded.

**Fig. 4:**
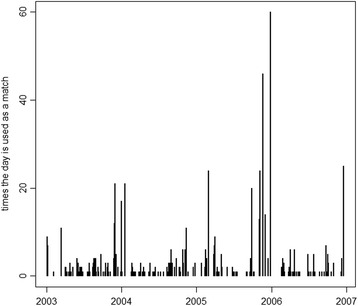
Number of times each control day is selected as a matched control, Milan, Italy, 2003–2006

Excluding control days never selected as matched controls (430), 46% of the remaining control days were selected once, 84% less than 5 times, 95% less than 15 times. Few days were selected a very large number of times (Fig. 4). The main consequence of using the same control day as a match for many times is an increase of the estimated variance, although with a benefit in terms of bias [41]. In order to get some insight on the influence of the control days used more than once as matches on the impact estimates, we investigated mortality and air pollution levels among those days, without finding any influential point. The control day that was used as a match for more than 60 treated days was characterized by a large number of deaths, thereby leading to a possible conservative lower impact.

Table 3 shows the results of the sensitivity analysis performed excluding the days of influenza epidemics from AD calculation. While AD for respiratory causes were substantially unchanged, the estimated impact on cardiovascular mortality among the elderly was lower, although still relevant (469 AD, 90% CI: 88, 850). We can conclude that our results were robust to possible bias derived from the confounding effect due to influenza epidemics.

**Table 3:** Estimated number of attributable deaths by cause and age class, after excluding influenza epidemic days, Milan, Italy, 2003–2006

	Age 15–64		Age 65–74		Age 75+		All ages (15+)	
	AD	90% CI	AD	90% CI	AD	90% CI	AD	90% CI
Cardiovascular causes	−78	−219, 63	93	−108, 294	469	88, 850	484	12, 956
Respiratory causes	−22	−91, 47	57	−7, 121	276	99, 452	311	122, 500
Other natural causes	28	−257, 313	16	−248, 280	−38	−434, 358	6	−514, 526
All natural causes	−72	−456, 312	166	−182, 513	707	100, 1314	801	6, 1595

*AD* attributable deaths, *90% CI* 90% confidence interval

## Discussion

Our analysis confirmed that having restrained PM_10_ levels under the EU limits could have avoided a relevant number of deaths in Milan during the study period, with an estimated impact even larger than the one reported in Baccini et al. [10], which was obtained following the standard approach based on Poisson regression, assuming a linear effect of air pollution on a logarithmic scale. ^1^ While we estimated a total of 1079 AD, Baccini et al. [10] found that exceeding the limits of 40 and 20 μg/m^3^ for PM_10_ annual average was responsible for 358 and 925 deaths from natural causes (89.5 and 231.3 per year), respectively. However, for a fair comparison we need to consider not only that the estimation methods were different, but also that the counterfactual scenarios were defined in a different way. While in the present analysis the counterfactual scenario consisted in daily levels of exposure lower than 40 μg/m^3^, with a resulting counterfactual annual PM_10_ average lower than 40 μg/m^3^ (31.6 μg/m^3^), the counterfactual scenario in Baccini et al. [10] was defined by fixing the counterfactual annual average exactly to 40/20 μg/m^3^.

Our approach relies on crucial assumptions. SUTVA requires that there are not hidden versions of the treatment and that the potential outcomes on one unit are unaffected by the specific treatment assigned to the other units (no-interference among units) [23]. In our context, this second condition could be critical, because the exposure in a day could affect mortality not only in the current day, but also in subsequent days. Focusing on the lag 0–1 exposure instead of on the current PM_10_ level makes the no-interference assumption more plausible. Obviously, enlarging the window of the moving average *X*_*i*_ would empower the no-interference assumption, but at the price of a lower variability of the exposures and of a reduced possibility of detecting an impact, if any. The other relevant assumption is the unconfoundedness assumption [35]. Being this condition not directly testable from the data, we grounded its plausibility on subject-matter knowledge derived from the literature. Note that we did not include individual level confounders in our analysis; as argued in Schwartz et al. [29], they are not relevant in this context, because we can reasonably assume their distribution to be rather stable on a day-to-day basis. We also implicitly assumed that there was no measurement error in PM_10_ levels. For causal inference with propensity score in the presence of treatment misclassification see, for example, Babanezhad et al. [43].

The idea of using matching is not new in the analysis of the short term effects of air pollution on health. The most popular example of matching in this field is the case-crossover approach, proposed as an alternative to Poisson regression with the aim of adjusting for the confounding effect of seasonality by design [44]. However, it is worth noting that the rationale of the propensity score matching is different from the rationale of the case-crossover approach. In the present analysis, we matched on the exposure variable by choosing for each high exposure day, the low exposure day exhibiting the closest propensity score. On the contrary, the case-crossover approach matches on the outcome: an individual who died in a certain day is matched with her/himself in one or more days when she/he did not die. Moreover, despite the use of matching, the case-crossover approach is more similar to a standard analysis based on Poisson regression, thus sharing its drawbacks and advantages [45], than to the approach proposed in this paper.

The approach we proposed has several advantages over the standard approach based on regression. The first one stems from the fact that it clearly distinguishes between the design phase and the analysis phase. The design phase (from propensity score estimation to matching) does not involve outcome data, but only background information. As a consequence, the sub-sample of units arising from the design phase (treated units and corresponding matched controls) can be used in the analysis phase to estimate the causal effects of the treatment on one or more outcomes (e.g. cause-specific and age-specific mortalities in our investigation). This also implies that results on different outcomes are fully consistent. For instance, the estimated total number of AD can be derived directly as sum of either age-or cause-specific AD. This consistency is not guaranteed within the standard model-based approach, as impact estimation by age and cause of death is usually obtained by fitting separate regression models for each outcome. For these characteristics, the proposed approach is promising to detect susceptible subpopulations and perform surveillance focusing on very specific causes of death or diseases.

A second advantage regards results interpretation. Our approach imposes to explicitly define the assumptions needed for drawing inference on the causal quantities of interest. On the contrary, results from regression rely on strong assumptions that are often not explicitly stated, thus making causal interpretation of the results controversial. Moreover, by clearly specifying the critical assumptions, we can assess the consequences of their violation; for instance, we could apply methods to evaluate robustness of the results to possible violation of unconfoundedness (see Chapter 21 and 22 in [23] for a comprehensive review).

A third advantage is that our approach is free from issues concerning the exposure-confounders-mortality modeling and does not involve extrapolation. Standard approaches based on regression models, where adjustment for confounders is achieved by including them in the regression function, can heavily rely on extrapolation if covariate distributions are substantially apart, i.e. if there are regions of the covariate space with relatively few treated or relatively few control units. This limited overlap between treated and control units can bring to poor model fit and inappropriate extrapolations. By using the approach proposed here, these problems can be detected and addressed more easily. Indeed, propensity score methods involve a careful description and implementation of the study design phase, which includes the construction of a group of matched controls with covariate distributions similar to those of the treated units. Although this may sacrifice some external validity, implying that inferences (for example attributable fractions) are less likely to be valid for populations with characteristics that are different from those observed in the sample, it awards a strong internal validity to impact estimates.

In order to correctly interpret the results of our analysis, some other points deserve discussion. The research question we focused on is different from the one of the studies which draw inference on the association (possibly interpreted as causal) between different “doses of exposure” and mortality/morbidity levels, with the aim to estimate a continuous exposure-response curve. In this paper, we considered the treatment as binary after having defined an arbitrary, although substantive, threshold, as our aim was to estimate the causal impact attributable to air pollutant levels higher than the threshold versus hypothetical levels lower than the threshold, without imposing any specific restriction on the exposure-response relationship.

It is worth to notice that, due to the absence of restrictions on the exposure-response function, our approach, based on propensity score matching, is substantially different from estimating impacts from a regression model where the exposure effect is modelled by a dummy variable, i.e. assuming a piecewise constant exposure-response relationship having a jump at the threshold. Also, unlike standard procedures for health impact assessment^1^, the method proposed in this paper does not rely on the log-linearity of the exposure-response relationship. This is an advantage, as the linearity assumption could bring to under or overestimated impacts, even in case of mild nonlinearity [11].

Our analysis did not provide an explicit estimate of the exposure-response curve. However, investigating the shape of this relationship may be of interest. In particular, evaluating a continuous exposure-response relationship and investigating the existence of a “safe threshold” under which the air pollutant would not affect health, could have important regulatory implications. Several studies in the epidemiological literature have investigated the shape of the exposure-response curve in this field, indicating that the linear assumption usually provides a good approximation of the relation within the ranges of exposure observed in urban areas [11–14]. However, all these studies are based on regression approaches; thus, exploring the exposure-response relationship through appropriate causal methods for continuous treatment, as those based on the generalized propensity score [30, 46], could be useful for comparison purposes and could provide estimates of the curve avoiding pitfalls due to inappropriate extrapolation.

Finally, our approach allowed us to consider counterfactual scenarios defined on daily exposures, but not in terms of annual average concentration, although these could be of interest from a legal and regulatory standpoint.

## Conclusions

In this paper we focused on the causal impact that a hypothetical intervention setting PM_10_ levels under a pre-fixed threshold would have had on mortality in Milan during the time frame 2003–2004. The method we used allowed to explicitly infer the causal impact of this hypothetical intervention, thus changing to some extent the scientific question underlying the estimation of an exposure-response function. It provided estimated impacts in terms of attributable deaths through a direct comparison of daily potential outcomes, relying on propensity score matching.

The proposed approach has various advantages over the standard regression methods. In particular, due to the clear distinction between the design phase and the estimation phase of the analysis, this approach assures consistency of the results between overall and subgroups analyses. It thus seems promising when the aim is performing surveillance focusing on very specific causes of death or diseases, or on susceptible subpopulations. Moreover, as the propensity score matching is free from issues concerning exposure-confounders-mortality modeling and does not imply extrapolation, the proposed method enhances robustness and internal validity of the results. For the same reason, although appealing for the assessment of historical impacts, it is not appropriate to estimate future impacts. Nevertheless, it should be considered as a tool to evaluate internal validity of the regression-based results, before any use of the estimated associations for projections purposes.

## Abbreviations

AD: Attributable deaths
CI: Confidence interval
PM10: Particulate matter ≤10 μm in diameter
RCM: Rubin’s Causal Model
SUTVA: Stable Unit Treatment Value Assumption

## Footnotes

1 According to the standard procedure, under the assumption of a linear effect of the air pollutant on a logarithmic scale, the number of attributable deaths (AD) can be calculated according to the following formula [10]:

$$ A D= y-{y}_0= y\left(1-1/ exp\left( b\left( x- T\right) I\left( x> T\right)\right)\right), $$

where $y$ is the observed number of deaths, $x$ is the observed level of the air pollutant, $b$ is the estimated coefficient expressing the air pollutant effect, $T$ is the fixed threshold and $I\left( x> T\right)$ is an indicator function which is equal to 1 if $x> T$ and 0 elsewhere. The calculation can be performed at the daily level or at the annual level. In the first case, $x$ and $y$ are daily values and $T$ is a fixed daily threshold; in the second case, $x$ is the annual average level of the air pollutant, $y$ is the annual number of deaths and $T$ is a fixed threshold defined on $x$.

2 Let **Z**_*i*_ denote the vector of the observed covariates for day *i*. The treatment assignment mechanism is strongly ignorable if the following conditions hold [35]: (i) Unconfoundedness: Treatment assignment is independent of the potential outcomes conditional on the observed covariates: *W*_*i*_ ⊥ (*Y*_*i*_(0), *Y*_*i*_(1))|**Z**_*i*_; (ii) Overlap: 0 < *P*(*W*_*i*_ = 1|**Z**_*i*_ = **z**) < 1 for each *i*. Because we are only interested in the impact for treated days, we can weaken the strong ignorability assumption as follows: (i) Unconfoundedness for controls: Treatment assignment is independent of the potential outcome under control conditional on the observed covariates: *W*_*i*_ ⊥ *Y*_*i*_(0)|**Z**_*i*_; (ii) Weak overlap: *P*(*W*_*i*_ = 1|**Z**_*i*_ = **z**) < 1 for each *i*. Unconfoundedness requires that there are no unobserved confounders, so that conditioning on the observed covariates assures that an experimental-like context is reproduced. The overlap assumption requires that there is sufficient overlap in the joint distribution of the covariates between treated and control days. This second assumption is needed because, if all days with given background characteristics had exposure higher than the threshold (*P*(*W*_*i*_ = 1|**Z**_*i*_ = **z**) = 1 for some **z**), then there would be no similar control days against which to compare them.

3 Pre-and post-matching standardized mean differences are defined as follows:

$$ {\delta}^{pre}=\frac{{\overline{x}}_{treated}-{\overline{x}}_{control}}{\sqrt{\frac{s_{treated}^2+{s}_{control}^2}{2}}}\kern0.5em \mathrm{and}\kern0.5em {\delta}^{post}=\frac{{\overline{x}}_{treated}-{\overline{x}}_{matched}}{\sqrt{\frac{s_{treated}^2+{s}_{control}^2}{2}}} $$

where ${\overline{x}}_{treated}$, ${\overline{x}}_{control}$ and ${\overline{x}}_{matched}$ are the sample averages of the covariate for treated units, controls and matched controls, and *s*_*treated*_ and *s*_*control*_ are the within-group sample standard deviations for treated and control units.

4 The estimate of the variance of $\widehat{AD}$ was derived from the sample variance proposed by Abadie and Imbens [40, 41] for the estimate of the Sample Average effect of Treatment on the Treated (SATT) estimator:

$$ {s}_{AD}^2={\displaystyle \sum_{i=1}^N{\left({W}_i-\left(1-{W}_i\right){K}_M(i)\right)}^2{\sigma}_{W_i}^2\left({\mathbf{Z}}_{\mathbf{i}}\right)}, $$

where *K*_*M*_(*i*) is the number of times the unit *i* is used as a match, and ${\sigma}_{W_i}^2\left({\mathbf{Z}}_i\right)$is the conditional variance of the outcome. The conditional variance can be estimated accounting for possible heterogeneity according to the following equation:

$$ {\sigma}_{W_i}^2\left({\mathbf{Z}}_i\right)=\frac{1}{\#{\mathrm{H}}_M(i)}{{\displaystyle {\sum}_{j\in \left\{{H}_M(i){\displaystyle \cup ( i)}\right\}}\left({Y}_j-{\overline{Y}}_{H_M(i){\displaystyle \cup ( i)}}\right)}}^2, $$

where *H*_*M*_(*i*) is the set of indexes for the first *M* matches for unit *i*, # *H*_*M*_(*i*) is the number of elements of *H*_*M*_(*i*) and ${\overline{Y}}_{H_M(i){\displaystyle \cup (i)}}$is the mean of the outcome in the set including the unit *i* and its matches. It is worth noting that the variance estimator ${s}_{AD}^2$does not account for the fact that the propensity score is estimated. The previous two equations are valid for each *M* ≥ 1; in our analysis we set *M* = 1.
